# Analysis of Granulometric Composition of Algal Suspensions in Wastewater Treated with Hydroponic Method

**DOI:** 10.1007/s11270-017-3556-5

**Published:** 2017-09-06

**Authors:** Aleksandra Bawiec, Katarzyna Pawęska, Krzysztof Pulikowski

**Affiliations:** Institute of Environmental Engineering, Wrocław University of Environmental and Life Sciences, 24 Grunwaldzki Sq., 50-363 Wrocław, Poland

**Keywords:** Algal suspensions, Wastewater treatment, Granulometric composition, Laser granulometer, Particle size analysis

## Abstract

The aim of the study was to determine the changes in suspension particle size identified in biologically treated wastewater, which was then treated in hydroponic system with use of engineering lighting by the light-emitting diodes (LED). The study was subjected to wastewater purified under laboratory conditions, in a hydroponic system using the effect of macrophytes *Pistia stratiotes* and growing algae. Measurement of particle size was made using a laser granulometer. Analysis of the results showed that the additional lighting of the hydroponic system with LED can significantly influence the ability of the suspension particles to agglomerate and, consequently, determine their sedimentation properties. In hydroponic system supported by additional lighting, more particles were observed with equivalent diameter *D*(3.2) smaller than 10 μm than those in the tank without additional lighting, indicating a higher reactivity of the particles. Determining the size of equivalent diameters *D*(4.3) allowed us to observe that in hydroponic system, particles of relatively small size predominate, which negatively affects the sedimentation process of the suspensions. Determination of particle size of suspensions consisting mainly of algae and the dynamics of their changes are the basis for specification of an effective method of removing particles from the system to protect the receiver from excessive suspension concentrations.

## Introduction

Irrespective of the type of wastewater treatment plant—its size or used technology, one of the main pollutants removed in the wastewater treatment process is suspensions. Reducing the amount of suspended solids in addition to the requirements for BOD_5_ and COD and the content of biogenic compounds is one of the basic requirements for wastewater treatment plants. Discharging into the surface water of sewage not sufficiently purified of organic and mineral particles can lead to disturbance of the natural ecological balance of the aquatic ecosystem (Reddy et al. 2014). In extreme cases, accumulation of suspensions in bottom sediments occurs, which may lead to a decrease in the depth of the receiver and changes in flow conditions (Burszta-Adamiak et al. 2011; Walters et al. 2014). Suspensions can also be transmitted over long distances, posing a threat to hydrodynamic processes in marine ecosystems (Liu et al. 2014; Dąbrowska et al. 2016).

The presence of colloidal and settleable suspensions in the outflow affects the effectiveness of the purification processes (Huang et al. 2010). Colloidal particles, by their construction, are the carrier of many impurities that threaten the quality of surface water (Rosse´ and Loizeau 2003). Substances containing heavy metals are readily adsorbed by colloidal particles. As a result of the sedimentation process, the metals adsorbed on suspension particles are transferred to bottom sediments, threatening the ecosystem of the river or lake (Yin et al. 2011; Suresh et al. 2012). This is particularly important for aquatic organisms because the composition and structure of bottom sediments determine their ability to settle (Remo et al. 2016). A significant aspect of wastewater treatment is the fact that the organic compounds associated with the grains of the suspensions can be significantly responsible for the COD values. One of the COD fractions itself is undissolved organic matter (Smyk et al. 2015), which leaves the treatment system with the outflow. The suspended solid sedimentation allows to some extent to remove organic matter from technological process when its residues from the purified sewage are immobilized in the bottom sediments of the receiver and undergo decomposition processes.

Effective removal of suspensions from sewage requires knowledge of the nature of the removed particles. Both shape and size are of great importance (Ma et al. 2001) because they affect the strength of the particles in aqueous solution (Bourcier et al. 2016). The effectiveness of commonly used wastewater treatment processes such as sedimentation, flocculation, coagulation, or disinfection is dependent on the nature of the suspensions (density, size, porosity, shape, and roughness of the particles) (Zielina 2016). It is therefore important to carry out an analysis of particle size distribution in wastewater and especially in systems using natural purification processes such as hydroponic systems.

Hydroponic purification systems are used in the three-stage wastewater treatment processes. After mechanical (screens, sieves, grit chambers) and biological (activated sludge reactors, hybrid reactors) cleaning, the wastewater is treated in hydroponic tanks, where the great importance has activity of algae, macrophytes, microorganisms, and organisms which occur also in natural river ecosystems. Organisms living in the tanks are responsible for reducing nitrogen and phosphorus loads flowing into the third stage of purification from the secondary settling tank. The idea behind such systems is to discharge to the receivers the wastewater of better quality than required by the law and sometimes even better than the water quality in the receiver. Macrophytes can have a major impact on the quality of surface water. Water plants that are in good condition help maintain high quality of water, and their dying results in intensive algae growth and deterioration of water quality, particularly in terms of turbidity (Olsen et al. 2015). The growth of algae in hydroponic systems is considered a desirable phenomenon, and algae-based wastewater treatment processes are termed phycoremediation. Biomass of algae is capable of taking nitrogen and phosphorus compounds from wastewater (Boelee et al. 2012).

For the correct functioning of the hydroponic system as a process of cleaning sewage from biogenic compounds, it is necessary to maintain the appropriate temperature and sunlight required by the macrophytes (Buzby and Lin 2014). In temperate climates, as the one defined in Poland, there is a clear seasonality. Hydroponic systems under such climatic conditions are built exclusively as indoor facilities. Walls surrounding hydroponic lagoons are provided with windows and skylights or are constructed of cellular polycarbonate to limit the impact of unfavorable temperatures on the purification processes and to provide as much sunlight as possible for the process of photosynthesis. In order to reduce the costs associated with hydroponic lagoon heating, in the less sunlit months, plant lighting systems can be used to extend the vegetation period and increase the efficiency of nitrogen and phosphorus removal. Providing light of a certain wavelength for the development of chlorophyll *a* improves the condition of the plant and supports the algae growth.

One of the major problems common in algae-based systems is their removal from the system after the purification process (Garbowski et al. 2017). Microalgae are one of the fractions of organic suspensions, and their removal from the wastewater treatment system results in an increase in the concentration of suspended solids at the outflow. The use of hydroponics in the sewage treatment process can on the one hand promote the removal of biogenes and, on the other hand, be the reason of the increased outflow of organic matter. Determination of particle characteristics in wastewater discharged from the hydroponic system should be the basis for the selection of system which can catch suspended solids and protect the receiver from contamination.

Determination of total suspended solid concentration is usually done using the weighing method and for the settling suspensions—a volume method. Both of these methods are indirect and time-consuming (Łomotowski et al. 2006). The determination of the amount of suspensions using traditional methods does not allow us to determine the percentage of particles of a certain size in the volume of tested solution or in the total number of identified particles. In the case of wastewater treatment plants using hydroponic systems, knowing the size of outflowing particles can help to identify their nature (organic, mineral, colloidal, clay fractions) and thus the choice of technology for their final disposal before discharge to the receiver. A solution for this type of technology may be the use of particle size distribution analysis using a laser granulometer.

The paper focuses on the use of modern analytical techniques, i.e., laser granulometry, for analyzing changes in suspension particle size and their equivalent diameters in sewage treated in a hydroponic system with additional lightening to intensify purification processes. The key task was to determine the ability of suspension particles composed mainly of algae for sedimentation and their removal from the system with use of conventional methods.

## Materials and Methods

### Samples Collection

**Fig. 1:**
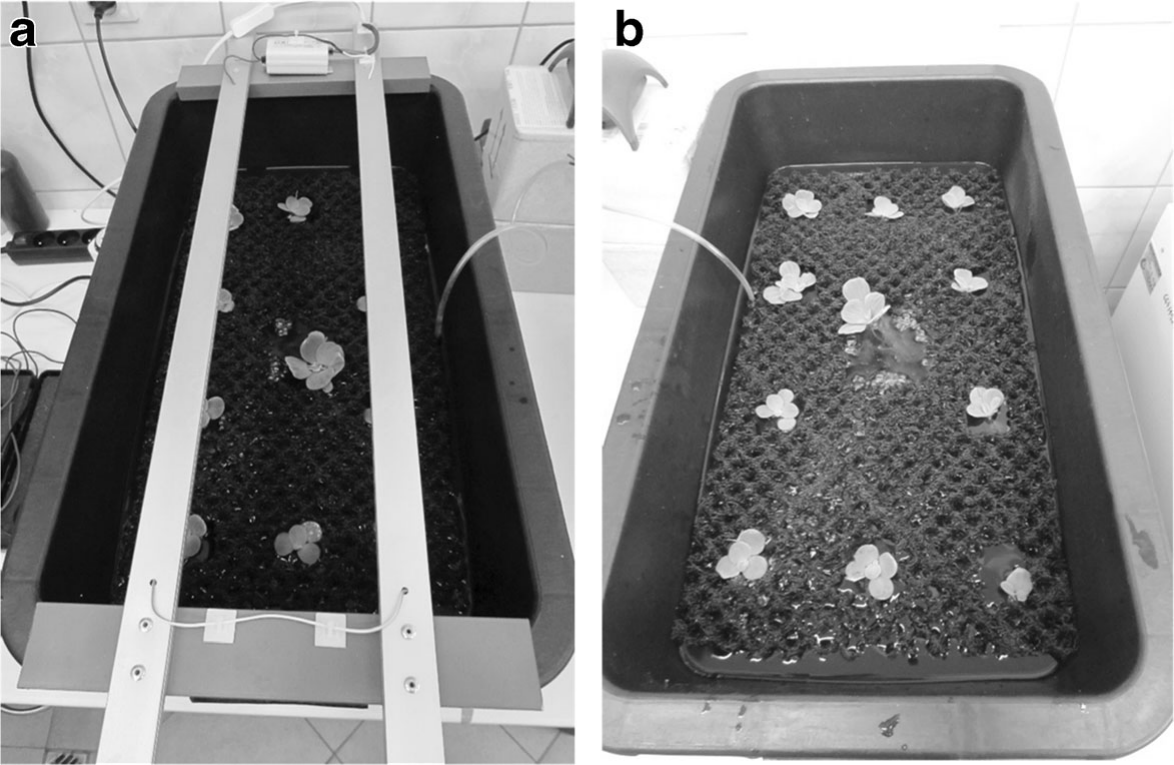
Experimental installation. **a** Macrophytes illuminated with LED. **b** Macrophytes without illumination

Samples of tested wastewater were collected from reservoirs simulating hydroponic lagoons placed in the Technological Laboratory of the Institute of Environmental Engineering, Wrocław University of Environmental and Life Sciences. Reservoirs of opaque walls, with a capacity of 60 dm^3^, were filled up to half (30 dm^3^) with biologically purified sewage taken from the inflow to the hydroponic lagoon from the municipal sewage treatment plant located in the Opole province. The tanks were aerated and planted with hydrophytes *Pistia stratiotes* used in the wastewater treatment plant. On one tank, a rack with LED light in the form of two rails with blue (450 nm) and red (620 nm) LEDs was installed. On each rail, the LEDs were in 4:2 ratio (red light:blue light). The exposure time was set from dusk to dawn (in the winter time from 5 pm to 8 am). The experimental installation is shown in Fig. 1a and b.

Each time, a sample was taken from an irradiated and unexposed tank in a volume of 800 ml to test the granulometric composition of wastewater. Measurements were made throughout the measurement series. Granulometric analysis was made on samples from three batches, each of which began with the introduction of biologically treated wastewater (after the secondary settling tank) from municipal wastewater treatment plant.

### Particle Size Measurement

Laser diffraction is a relatively quick method of determining the amount of suspensions in aqueous solutions as well as the particle size distribution (Ma et al. 2001). The measurements were made using the Malvern Mastersizer 2000 laser granulometer to determine the percentage of particles in the size range of 0.02–2000 μm. The principle of this device operation is based on the interaction of three elements: red and blue light sources, measuring cells, and detectors. The emitted laser beam is dispersed onto the grains of the suspensions flowing into the measuring cell solution. Detectors measure the intensity of the light scattered on the particles and allow them to be identified by their size. The test solution is delivered to a measuring cell using a Hydro MU dispersion paddle with paddle stirrer, which makes the solution homogeneous (Sperazza et al. 2004; Kuśnierz and Łomotowski 2015).

## Results

**Fig. 2:**
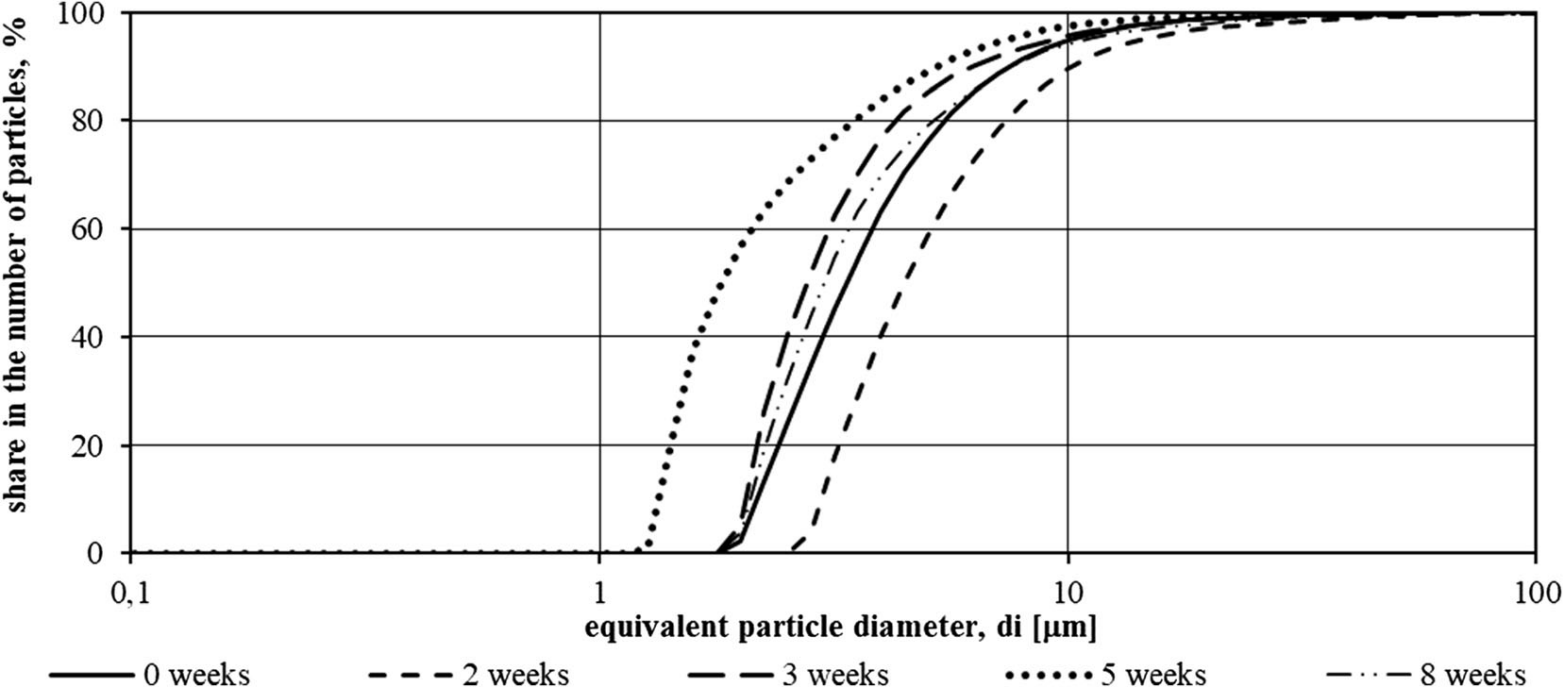
Function *F*(*di*) of suspensions specified in samples from the tank illuminated with LED in the period from 15.11.2015 to 8.01.2016 (first measurement series—8 weeks)

**Fig. 3:**
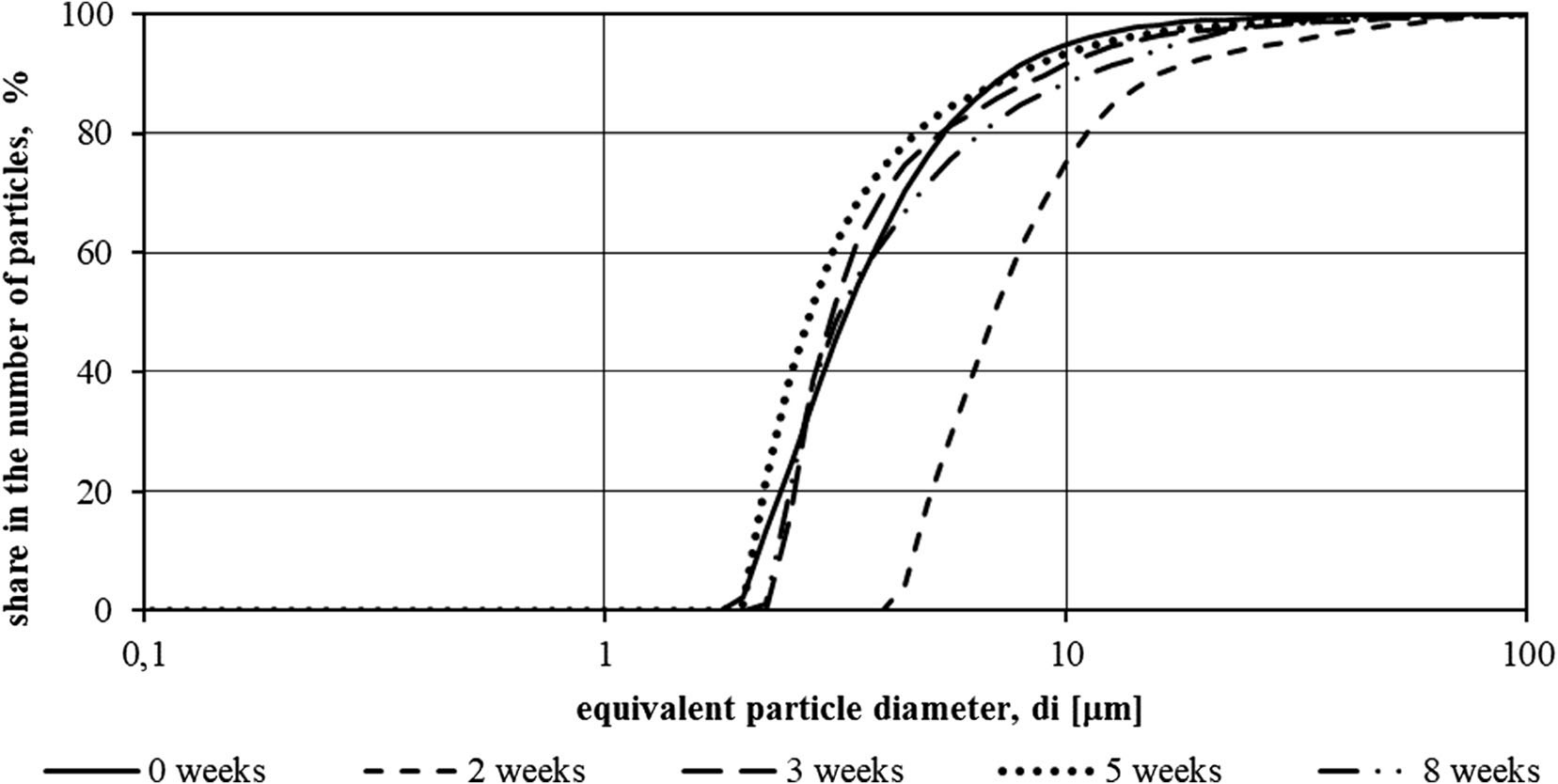
Function *F*(*di*) of suspensions specified in samples from the tank without LED illumination in the period from 15.11.2015 to 8.01.2016 (first measurement series—8 weeks)

Studies made with use of a laser granulometer allowed to define the range of particle size of suspensions present in the tank with LED lighting and without additional light. Results are presented as percentages of amount of particles with *di* diameter using the *F*(*di*) function distributions. Cumulative plots of particle size distribution in samples taken from the illuminated tank and tank without additional light source for the first measurement series are shown in Figs. 2 and 3.

During the first measurement series, i.e., from 15.11.2015 to 8.01.2016, both in the wastewater from the hydroponic tank using the illumination and those from the second tank, the particles showed a wide range of sizes. The lower limit of the particle size range was 0.1 μm, with the upper reaching 100 μm.

In the case of particles identified in the lighted sewage, a rise in particle size was observed after a week of study and then decreased until the fifth week of study. In the last 2 weeks of study (from 18.12.2015 to 8.01.2016), a particle size increase was observed—the particle size distribution curve approached the particle distribution curve in the wastewater filled into the tank at the beginning of the measurement series.

In wastewater treated without artificial light, a similar tendency for growth and decrease in particle size was observed, but a re-growth of their dimensions was observed earlier than in the case of illuminated sewage. At the end of the experiment, the particle dimensions approached the dimensions of biologically purified wastewater introduced into the reservoirs on 15.11.2015.

**Fig. 4:**
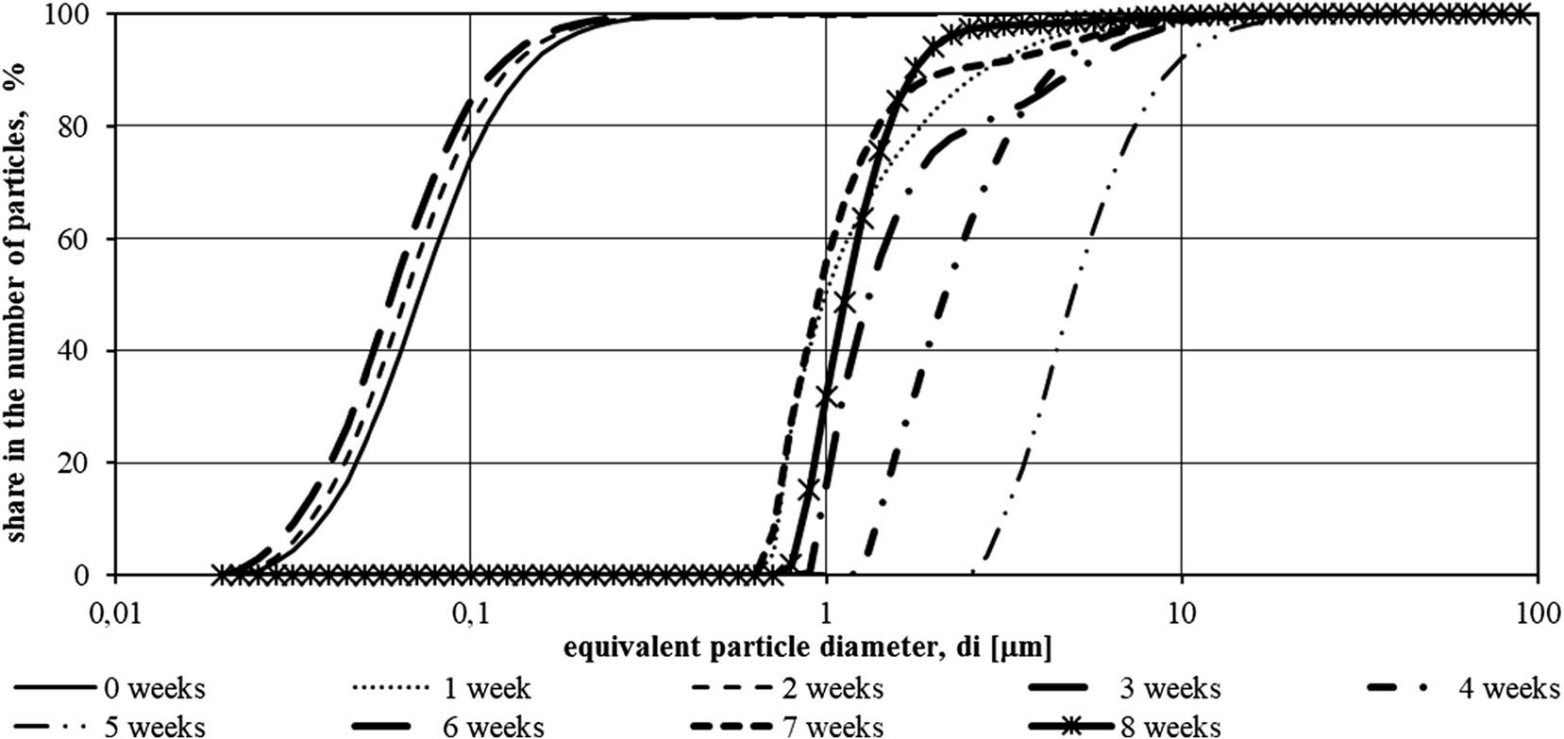
Function *F*(*di*) of suspensions specified in samples from the tank illuminated with LED in the period from 15.01.2016 to 11.03.2016 (second measurement series—8 weeks)

**Fig. 5:**
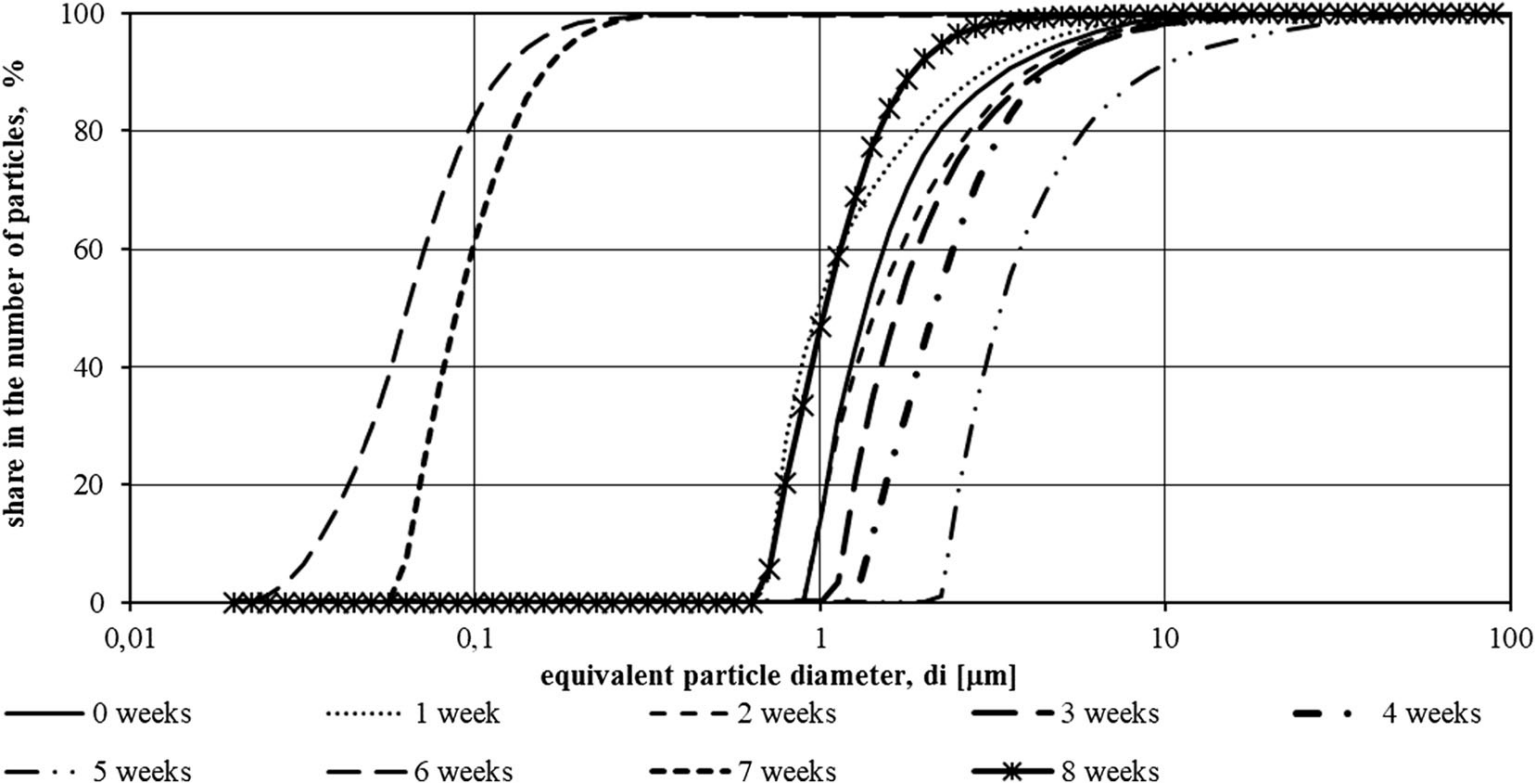
Function *F*(*di*) of suspensions specified in samples from the tank without LED illumination in the period from 15.01.2016 to 11.03.2016 (second measurement series—8 weeks)

Figures 4 and 5 show cumulative plots of particle size distribution in the total amount of suspensions, obtained from granulometric tests of wastewater from the second measurement series.

The particle size of slurries in the wastewater tested during the second measurement series (from 15.01.2016 to 11.03.2016) in both tanks was in the range of 0.02–100 μm.

In the sewage collected from the tank using additional red and blue lighting, an increase in particle size was observed in the first week of experiments. From January 22, 2016 (1 week of experiment), a decrease in the size of the particle diameters was observed over the next 2 weeks, followed by an increase to from third to fifth weeks of the study. After another 2 weeks of experimenting, there was a further decrease in the number of particles with larger *di* diameters.

In the case of tank with wastewater without additional light source, changes in particle size distribution were characterized by different dynamics. In the first week of the experiment, the particle size decreased, but for the next 4 weeks, they were characterized by successive growth. In the week of 19.02.2016 to 26.02.2016 (from fifth to sixth week), a decrease in the particle diameters of the suspensions occurred and then another increase in the last 2 weeks.

**Fig. 6:**
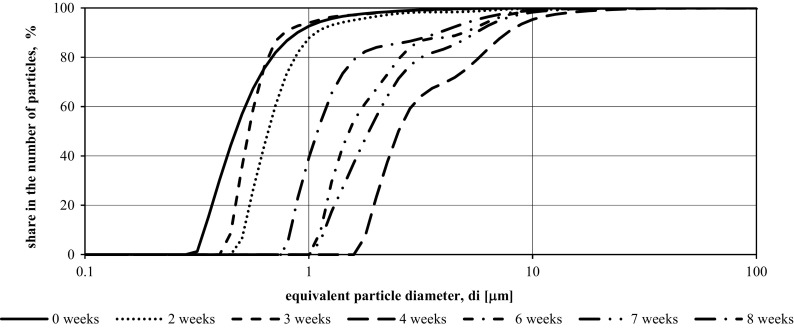
Function *F*(*di*) of suspensions specified in samples from the tank illuminated with LED in the period from 8.04.2016 to 30.05.2016 (third measurement series—8 weeks)

**Fig. 7:**
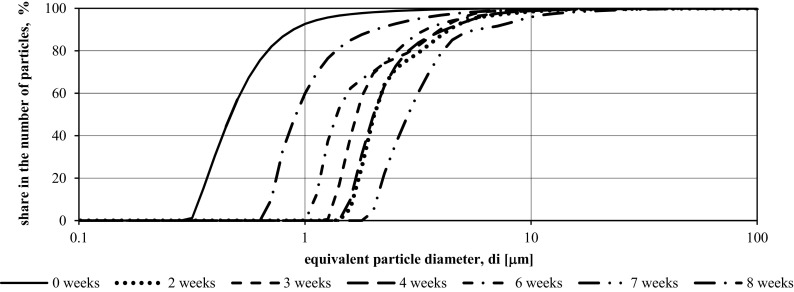
Function *F*(*di*) of suspensions specified in samples from the tank without LED illumination in the period from 8.04.2016 to 30.05.2016 (third measurement series—8 weeks)

Figures 6 and 7 with cumulative particle size distribution charts show the results of granulometric tests of illuminated and underexposed wastewater in the third measurement series.

The particle size ranges present in the illuminated and un-illuminated effluents were the same—0.1–100 μm. Changes in particle size in the tank equipped with the lighting system started with a significant increase in the size of the suspensions in the first 2 weeks of the experiment. After April 15, 2016, particle diameters decrease for 2 weeks followed by an increase in the fourth week. In the last two weeks of the measurement series, successive decreases and increases in the diameter of suspensions contained in wastewater have been observed.

In the tank without additional lighting, the change in diameters was different in dynamics than that in the tank equipped with LED installation. The increase in particle size was observed for 2 weeks, followed by an alternation of diminishing and increasing *di* diameters in each subsequent week.

Changes in the size of equivalent diameters in both the illuminated and underexposed tanks were due to the development of algae. The development of algae in tanks was characterized by varying intensities resulting from different environmental conditions. As the algae grew, changes in hydrobiological composition were noted—with the passing of time, strong diatom species were developed excluding other species.

The temperature of surrounding air in the first and the second measurement series was nearly the same and varied from 15 to 20 °C. The temperature of the wastewater in both illuminated and underexposed tanks in the first measurement series varied from 12.7 to 19.3 °C and in the second measurement series from 13.6 to 19 °C. In the third measurement series (from 8.04.2016 to 30.05.2016), the temperature of wastewater was slightly higher and during the time of experiment varied from 16.2 to 21.4 °C, because of the higher temperatures of surrounding air that was in range from 16.5 to 22 °C.

Each of the series began with the introduction into the tanks of biologically treated sewage collected from municipal sewage treatment plant. Small colonies or single algae cells were present in wastewater. The increase in particle size during the initial study periods was related to the increased algae multiplication and the growth of the green algae colonies observed under the microscope. Over time, the green algae colonies were dispersed and, in the tank, dominated the diatoms in the form of individual organisms or clusters settled on sediment flocs, dead plant fragments, or substrate particles.

For wastewater treatment processes, excessive algae growth in the purification process may cause secondary contamination and discharge of higher suspended solid concentrations to the receiver. Studies of granulometric composition of sewage allow to determine the equivalent particle diameters describing their structure: *D*(1.0)—average length, *D*(2.0)—average surface, *D*(3.0)—average volume, *D*(3.2)—the size of the equivalent sphere in terms of surface area (average size of surface area of the particle), and *D*(4.3)—the equivalent sphere in terms of the volume (the location of the gravity center around which the remaining particles are concentrated) (Baliński 2013). Analysis of particle size, surface area, average diameter, or volume may be helpful in determining the optimum moment in which the discharge of wastewater to the receiver would not result in excessive outflow of suspended solids. Knowing the size of particles before their discharge may also be helpful in analyzing the sedimentation properties of suspensions and the choice of technology for separating excess algae from wastewater discharges.

**Fig. 8:**
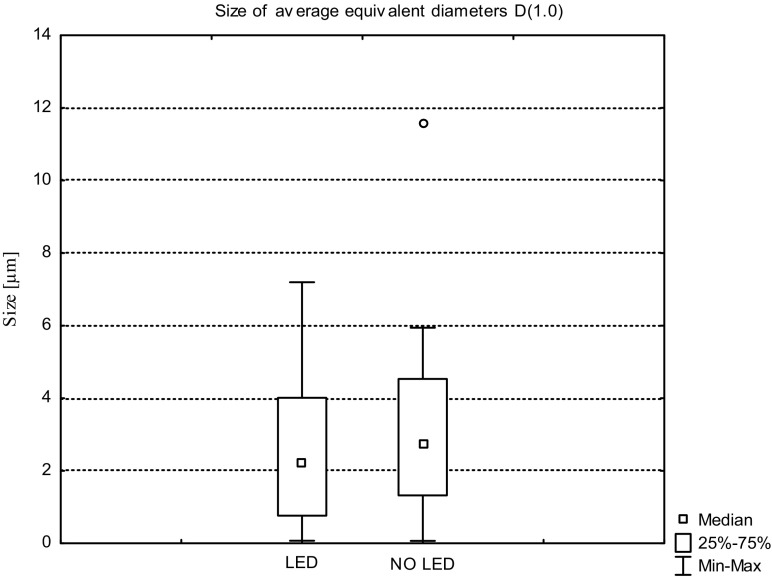
Sizes of the average equivalent diameters of suspensions *D*(1.0) in sewage taken from tank with LED and without LED lighting, determined during the three measurement series

**Fig. 9:**
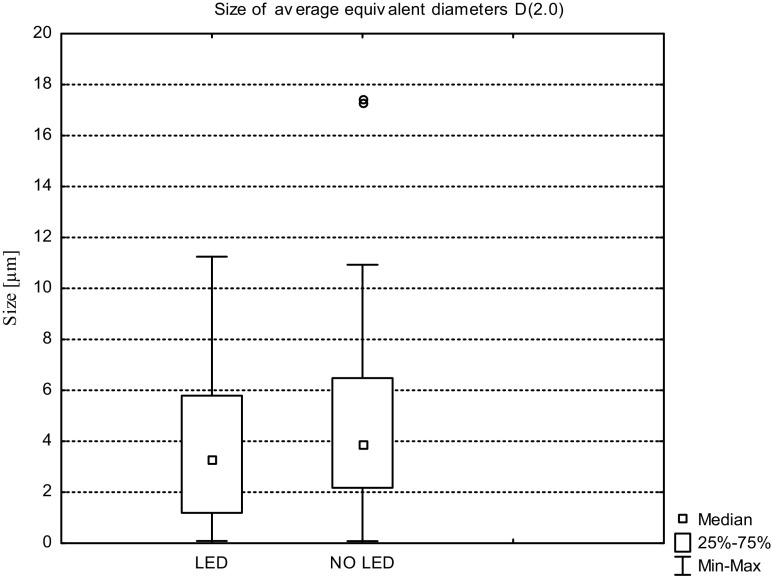
Sizes of the average equivalent diameters of suspensions *D*(2.0) in sewage taken from tank with LED and without LED lighting, determined during the three measurement series

**Fig. 10:**
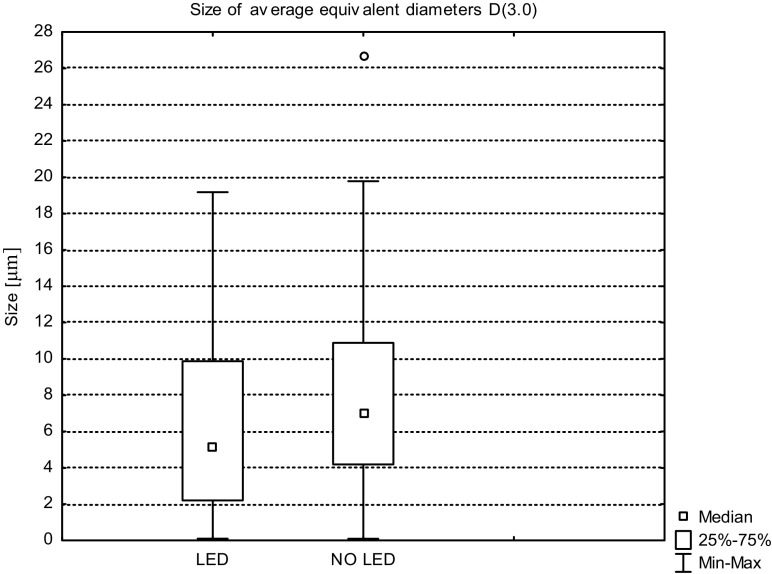
Sizes of the average equivalent diameters of suspensions *D*(3.0) in sewage taken from tank with LED and without LED lighting, determined during the three measurement series

The average values of *D*(1.0), *D*(2.0), and *D*(3.0) for the wastewater from the illuminated tank and from reservoir without additional lighting obtained during the three measurement series are shown in Figs. 8, 9, and 10.

The size of the equivalent diameter *D*(1.0) of suspended solid particles in the wastewater gives an information about size of particles that prevails in polydisperse suspensions (Wiercik et al. 2016). Analysis of the size distribution of particle *D*(1.0) diameters showed that in the case of illuminated sewage, the range of occurring particle sizes is wider than in the case of effluent without illumination. Particles of suspensions collected from the tank, with LED-assisted purification, have a diameter *D*(1.0) in the range of 0.074 to 7.119 μm with a significant concentration of particles in the range of 1 to 4 μm. No extreme values were observed, whereas particle size distribution was characterized by significant asymmetry. In the case of effluents without additional lighting, the size of the average equivalent diameters of the particles was in the range of 0.070 to 5.994 μm. The median value for a given set of equivalent diameters *D*(1.0) was 2.743 μm. The diameter distribution was symmetrical with less dispersion than in the particles identified in the wastewater lighted with LED.

Analysis of the average particle surface area of suspensions expressed by size of average equivalent diameter *D*(2.0) showed that in the case of illuminated effluent as well as without an additional light source, the diameter size distribution is similar. In the wastewater from tank with LED, the size range of the equivalent diameters *D*(2.0) was 0.087 to 11.284 μm and 75% of the resulting value was within the range of 5.797 μm. The size distribution was asymmetric on the right; no outlier values were identified. The effluent from the reservoir without illumination was characterized by the presence of particle sizes *D*(2.0) in the range of 0.081 to 10.929 μm for which the median was 3.886 μm. The extreme value for a given set of diameters was 17.500 μm and occurred twice during the experiment—during the first measurement series (15.11.2015–8.01.2016). Size distribution was characterized by lower asymmetry than in case of the size of the particles identified in sewage with LED lighting.

The size of the equivalent diameter *D*(3.0) is measured by measuring the volume of each of the particle present in the solution and then dividing the values by the number of tested particles. Dimensions of average diameters in relation to volume *D*(3.0) both for suspensions in wastewater with and without additional light source were in the range of 0.120 to 19.500 μm and the distribution was asymmetric. For wastewater without LED lighting, extreme values were recorded during the first measurement series (27.11.2015).

The size of the *D*(3.2) and *D*(4.3) equivalent particle diameters obtained during the experiment (three measurement series) for both of the tanks is shown in Table 1.

**Table 1:** The sizes of the average equivalent diameters of suspension particles *D*(3.2) and *D*(4.3) in wastewater taken from experimental tanks, determined during the three measurement series

Date	Tank with additional LED light		Tank without additional LED light	
	*D*(3.2)	*D*(4.3)	*D*(3.2)	*D*(4.3)
First measurement series				
15.11.2015	30.647	67.705	30.647	67.705
27.11.2015	55.826	99.655	62.901	99.267
4.12.2015	47.722	89.327	64.813	101.401
18.12.2015	35.162	69.788	58.145	96.829
8.01.2016	37.383	64.698	29.242	49.935
Second measurement series				
15.01.2016	0.183	0.419	0.183	0.419
22.01.2016	18.663	44.364	19.640	43.901
29.01.2016	0.151	0.264	17.678	39.597
4.02.2016	0.141	0.190	20.360	44.799
12.02.2016	14.274	42.481	23.791	54.202
19.02.2016	19.125	51.203	44.546	75.780
26.02.2016	12.072	26.625	0.131	0.209
3.03.2016	13.619	31.870	0.201	0.648
11.03.2016	7.476	17.260	12.074	36.944
Third measurements series				
8.04.2016	3.965	9.645	3.965	9.645
22.04.2016	7.502	15.520	23.729	59.274
29.04.2016	5.277	12.436	20.618	49.866
6.05.2016	25.663	60.548	25.337	61.687
16.05.2016	12.966	33.018	25.551	64.039
23.05.2016	12.907	31.953	33.937	67.616
30.05.2016	11.087	23.381	15.551	31.117

The average diameter *D*(3.2) calculated from the volume ratio to the sum of the particle surface gives information about the size of the active surface. The smaller the diameter *D*(3.2), the surface of the particles is greater, which increases the efficiency of the particles in the catalysis of chemical reactions (Wiercik et al. 2016). Equivalent diameter *D*(4.3) is calculated on the basis of moment of mass and volume, giving an information about the mass concentration of the particles in the suspension. The size of *D*(4.3) diameter is determined by the size of large particles (Kuśnierz and Wiercik 2016).

The values of diameter *D*(3.2) in each measurement series were lower in the illuminated wastewater than in the effluents from the tank without LED light installation. During the entire experiment, the smallest and largest diameters were, however, recorded in the sewage from tank without additional light source and were respectively 0.131 and 64.813 μm. The minimum value was recorded in the second measurement series after 42 days of experimentation, while the maximum in the first series after 19 days. In the case of illuminated effluents, the minimum value was 0.114 μm, while the maximum reached 55.826 μm and was recorded in the first series after 12 days from the start of the experiment.

Diameter *D*(4.3) was characterized by values ranging from 0.190 to 99.65 μm in illuminated effluents and from 0.209 to 101.401 μm in effluents not illuminated by LED light. As with the *D*(3.2) diameter, higher values were noted for wastewater where no additional light was used. Maximal *D*(4.2) diameter values were obtained after 19 days of experiment in the first measurement series, but it should be noted that the *D*(4.3) diameter values in the wastewater introduced into the experiment exceeded 67.700 μm. In the other two measurement series, equivalent diameters *D*(4.3) in wastewater introduced into the experiment were less than 10,000 μm, and the maximum values for sewage without illumination were successively 75.780 μm in the second measurement series and 67.616 μm in the third series.

## Discussion

Analysis of the obtained results shows that in the hydroponic systems used to treat wastewater after biological treatment in a hybrid circulating biological reactor, the particle size of the suspensions ranges from 0.1 to 100 μm. Among organisms creating suspensions which sizes were identified during experiment, algae, including diatoms and green algae, as well as protozoa and other zooplankton and phytoplankton organisms can be distinguished (Round et al. 1990; Gianuca et al. 2016). Changes in the size of particles during the experiment, both during LED illumination and without the addition of an external light source, may indicate that their compositions have been altered, including the change in the nature of their dominant organisms. Most important for the quality of sewage from hydroponic systems is the content of algae developing in the solution that form the multidispersion suspensions, which makes it difficult to describe the size and shape of the particles (Rolinski et al. 2013). Cells of algae, depending on the species, form different shapes, may occur singly or in colonies, and their size can change by up to 100 μm (Stoyneva et al. 2007). Determining the dynamics of particle size changes that form the bulk of the algae suspensions and estimating the moment in which the suspension parameters are most beneficial for its effective removal from the system may be the basis for the development of new methods for wastewater treatment.

The sizes of equivalent diameters *D*(1.0), *D*(2.0), and *D*(3.0) measured during experiment, which gives an information about each particle dimension, give also the general information about their approximate shape. Most important in the process of transformation of wastewater during the purification process is, among others, the change in average diameter *D*(3.2) which determines the reactivity of the particles. As showed Wiercik et al. (2016), particles of suspensions with determined diameter *D*(3.2) less than 10 μm have a high absorption capacity. In the case of suspended structures identified in the sewage from experiment, average diameters *D*(3.2) in wastewater from tank with LED installation in 33% are those less than 10 μm. In wastewater taken from tank without additional lighting, only 19% of identified *D*(3.2) diameters had values that did not exceed 10 μm. Based on data presented in the work of Kuśnierz and Wiercik (2016), it can be concluded that in LED-treated wastewater during the entire experience of three measurement series, 14% of the particles were characterized by greater active area and thus better sorption properties than particles from sewage treated without LED. However, in both of the tanks, in each of the three measurement series, particles with diameters greater than 10 μm predominate, indicating their low catalytic capacity and poor reactivity.

Another parameter describing the nature of the suspension particles investigated during the experiment was the change in average diameter *D*(4.3), which was an indicator of particle mass. During the entire study period, the maximum identified average diameter *D*(4.3) was slightly over 100 μm. In the vast majority (71% of observations in illuminated effluents and 57% of observations in effluents without LED illumination), the values of diameters did not exceed 50 μm. Kuśnierz and Wiercik (2016) studies show that this is a relatively small value, which indicates that suspensions have low sedimentation capacity.

## Conclusions

Based on the analysis of the granulometric composition of suspensions in hydroponic systems—assisted by LED illumination and without artificial lighting—it can be concluded that the use of an additional light source in the system to support the photosynthesis influences the change in particle size of suspensions in wastewater.

Additional lighting of the sewage with macrophytic plants with 450 and 620 nm wavelengths resulted in smaller particle sizes than experiments conducted without an additional light source. The illumination process not only supports the growth of macrophytes and increases their viability but also significantly changes the composition of the suspensions, which can determine its sedimentation capacity and the possibilities for its removal from the system. Determining the optimal moment of removing suspended solids from the hydroponic system is a difficult task and requires further research on the granulometric composition of suspensions, their transformation in the aquatic environment, and the species composition of their organisms. It is also necessary to determine the nature of the processes that influence particle size change and their aggregation in LED-illuminated systems and without nighttime lighting, what is the subject of further research conducted by the authors.
